# Inter-species interactions between two bacterial flower commensals and a floral pathogen reduce disease incidence and alter pathogen activity

**DOI:** 10.1128/mbio.00213-24

**Published:** 2024-02-20

**Authors:** M. Amine Hassani, Zhouqi Cui, Jacquelyn LaReau, Regan B. Huntley, Blaire Steven, Quan Zeng

**Affiliations:** 1 Department of Plant Pathology and Ecology, The Connecticut Agricultural Experiment Station, New Haven, Connecticut, USA; 2 Department of Environmental Science and Forestry, The Connecticut Agricultural Experiment Station, New Haven, Connecticut, USA; University of Nebraska–Lincoln, Lincoln, Nebraska, USA

**Keywords:** *Erwinia amylovora*, *Pseudomonas*, *Pantoea*, emergent property, meta-transcriptome, co-culture, co-inoculation, fire blight, *Malus domestica*

## Abstract

Flowers are colonized by a diverse community of microorganisms that can alter plant health and interact with floral pathogens. *Erwinia amylovora* is a flower-inhabiting bacterium and a pathogen that infects different plant species, including *Malus × domestica* (apple). Previously, we showed that the co-inoculation of two bacterial strains, members of the genera *Pseudomonas* and *Pantoea,* isolated from apple flowers, reduced disease incidence caused by this floral pathogen. Here, we decipher the ecological interactions between the two flower-associated bacteria and *E. amylovora* in field experimentation and *in vitro* co-cultures. The two flower commensal strains did not competitively exclude *E. amylovora* from the stigma habitat, as both bacteria and the pathogen co-existed on the stigma of apple flowers and *in vitro*. This suggests that plant protection might be mediated by other mechanisms than competitive niche exclusion. Using a synthetic stigma exudation medium, ternary co-culture of the bacterial strains led to a substantial alteration of gene expression in both the pathogen and the two microbiota members. Importantly, the gene expression profiles for the ternary co-culture were not just additive from binary co-cultures, suggesting that some functions only emerged in multipartite co-culture. Additionally, the ternary co-culture of the strains resulted in a stronger acidification of the growth milieu than mono- or binary co-cultures, pointing to another emergent property of co-inoculation. Our study emphasizes the critical role of emergent properties mediated by inter-species interactions within the plant holobiont and their potential impact on plant health and pathogen behavior.

**IMPORTANCE:**

Fire blight, caused by *Erwinia amylovora*, is one of the most important plant diseases of pome fruits. Previous work largely suggested plant microbiota commensals suppressed disease by antagonizing pathogen growth. However, inter-species interactions of multiple flower commensals and their influence on pathogen activity and behavior have not been well studied. Here, we show that co-inoculating two bacterial strains that naturally colonize the apple flowers reduces disease incidence. We further demonstrate that the interactions between these two microbiota commensals and the floral pathogen led to the emergence of new gene expression patterns and a strong alteration of the external pH, factors that may modify the pathogen’s behavior. Our findings emphasize the critical role of emergent properties mediated by inter-species interactions between plant microbiota and plant pathogens and their impact on plant health.

## INTRODUCTION

The flower microbiota provides a useful model for studying the ecological processes of plant microbiome assembly (1). These short-lived plant organs support the growth of a diverse community of microorganisms (2, 3). The microbial communities that inhabit flowers convey many functions including interactions with pollinators, altering flower chemistry, and providing a potential defense mechanism against floral pathogens (1, 4). The inter-species interactions between plant microbiota and plant pathogens may lead to properties that mediate disease suppression (5, 6). Exploring these interactions within the plant holobiont could reveal new mechanisms to control plant diseases and promote plant health.

Flowers are nutrient-rich microbial habitats coveted by plant pathogens (7). *Erwinia amylovora* is a pathogen that infects several plant species of the *Rosaceae* family (8). This bacterium causes fire blight, which incurs significant losses in pome fruit production. Conventional management of fire blight relies mainly on the prophylactic application of antibiotics, such as streptomycin, oxytetracycline, or, to a lesser extent, oxolinic acid or kasugamycin (9). However, these management strategies lead to the evolution of antibiotic resistance in *E. amylovora* (9). The emergence of antibiotic-resistant strains drives pressure to develop sustainable practices to control fire blight. Microbial inoculants offer alternative strategies to protect plants against pathogens. Examples include the bacterium *Pantoea agglomerans*, known to secrete antibiotics that inhibit *E. amylovora* in the field (10), *or Pantoea vagans*, which excludes the pathogen from the stigma by competing for limiting substrates (11, 12). Lytic phages have also been shown to protect against the fire blight disease (13). While the biological control of the fire blight by these microbial species is mainly mediated by competitive niche exclusion, direct antagonism, or competing for space and nutrients, little consideration is given to the role of inter-species interactions beyond competition that mediate the suppression of plant diseases (14).

In an earlier study, we showed that the co-inoculation of *Pseudomonas* CT-1059 (*Ps*) and *Pantoea* CT-1039 (*Pa*), two bacterial strains isolated from apple flowers, leads to reduced disease incidence in experimental orchards (15). These two microbiota members did not show evidence of strong inhibitory interactions against *E. amylovora* in culture, and disease suppression was highest in the presence of both inoculated strains, suggesting that ecological interactions among the members likely accounted for the suppression of the fire blight disease (15). In the present study, we aimed to decipher the ecological interactions between the two flower-associated bacteria (i.e., *Ps* and *Pa*) and the fire blight bacterium *E. amylovora* using field co-inoculations and laboratory co-culture growth conditions. We hypothesized that bacterial inter-species interactions may alter pathogen activity and promote plant health. To this end, we investigated the potential of co-inoculation of *Ps* and *Pa* to influence the establishment, survival, and activity of the pathogen *E. amylovora*. Using synthetic stigma exudation medium (16), we characterized how strain co-cultures altered bacterial growth and gene expression. This study demonstrates that ecological interactions between microbiota commensals and plant pathogens are inherently complex and could lead to emergent functions with significance to plant health.

## RESULTS

### Co-inoculation of two flower bacterial commensals reduces fire blight occurrence

**Fig 1:**
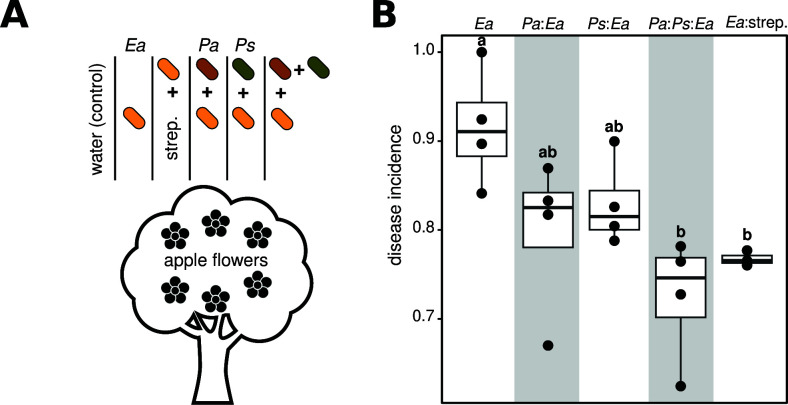
Co-inoculation of two flower commensal bacteria reduces disease incidence in experimental orchards. (A) The drawing shows the experimental procedure of co-inoculating *Pseudomon*as and *Pantoea* on apple flowers “Red Delicious.” Briefly, apple trees were either inoculated with water (control), with *Ea*, with *Ea* than treated with streptomycin (*Ea*:strep), inoculated with *Pantoea* (*Pa*) or *Pseudomonas* (*Ps*) than treated with *Ea (Pa:Ea* or *Ps:Ea*, respectively), or co-inoculated with *Ps* and *Pa* then treated with *Ea* (*Pa:Ps:Ea*). (**B)** Boxplots show fire blight disease incidence in the field under the five treatments: *Ea*, *Pa:Ea*, *Ps:Ea*, *Pa:Ps:Ea*, and *Ea:s*trep. Co-inoculation of the flowers with *Pseudomonas* and *Pantoea* significantly reduced fire blight incidence in the experimental field. Each circle corresponds to one tree; the letter indicates significant differences.

We showed previously that the probiotic co-inoculation of two flower-associated bacteria, *Ps* and *Pa*, reduces fire blight occurrence (15). The inoculation of these strains was repeated in this study (Fig. 1A; Materials and Methods). In brief, four trees of the apple cultivar “Red Delicious” were treated either with water (control), only the fire blight pathogen *Erwinia amylovora* 110 (*Ea*), *Pa* or *Ps* followed by the inoculation of *Ea* (*Pa:Ea* or *Ps:Ea*, respectively), or mixture of *Pa* and *Ps* followed by *Ea* inoculation (*Pa:Ps:Ea*). The co-inoculation of both flower commensal bacteria significantly reduced fire blight incidence to a similar extent as the streptomycin treatment (*Pa:Ps:Ea* vs *Ea*:strep., respectively), whereas single isolate inoculations of *Pa* or *Ps* at the same cell concentration (either *Pa:Ea* or *Ps:Ea*) did not significantly reduce the disease incidence (Fig. 1B). These results verify our previous findings and show that the co-inoculation of these two flower commensal bacteria significantly reduce disease incidence in field experimentation, indicative of the role of ternary interactions between the species and the potential emergence of a microbial-mediated plant phenotype.

### The pathogen *E. amylovora* remains abundant and active in apple flowers

**Fig 2:**
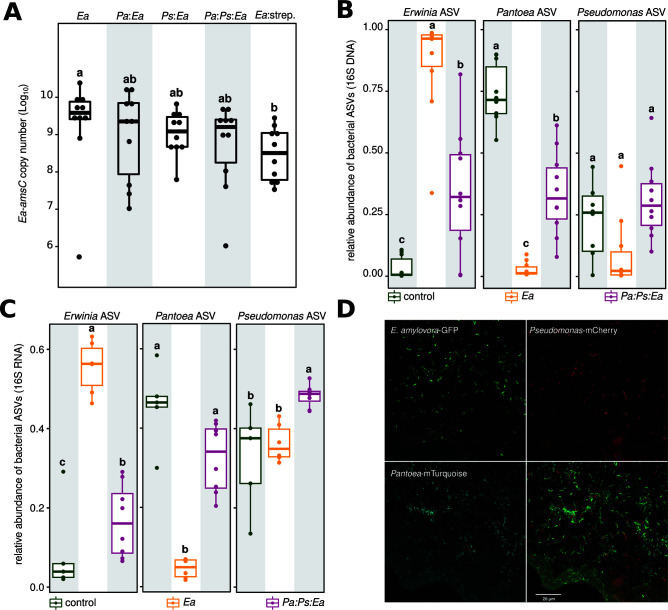
The two flower commensal bacteria do not competitively exclude *E. amylovora* from the stigma. (A) The boxplots depict *amsC* gene copy number of *E. amylovora* on the flower stigma. Each circle corresponds to one flower, and letters indicate significant differences between treatments. *E. amylovora* was detected in all treatments. None of the inoculations significantly reduced the population size of *Ea*, except the application of streptomycin. (**B** and **C)** The boxplots show the relative abundance of *Erwinia* amplicon sequence variant (ASV), *Pantoea* ASV, and *Pseudomonas* ASV revealed by amplicon sequencing of the 16S gene and gene transcript, respectively. Although co-inoculation of *Pa* and *Ps* reduced the relative abundance of *Ea*, the 16S gene (**B**) and gene transcript (**C**) of *E. amylovora* were readily detected in all treatments. (**D)** Micrographs show confocal microscopy of *E. amylovora* expressing Green Fluorescence Protein (GFP) (top left), *Pseudomonas* expressing mCherry (top right), *Pantoea* expressing mTurquoise (bottom left), and the overlay of the three channels (bottom right) on the stigma of apple flower. *Ps* and *Pa* were first inoculated on the stigma, then *Ea* was applied after 1 day. Microscopy pictures were acquired 2 days after the inoculation of *Ea*. All three bacterial strains are detected and express fluorescent proteins on the stigma of apple flowers.

To investigate whether the flower commensal strains (i.e., *Pa* and *Ps*) exclude *E. amylovora* from colonizing the apple flowers, we quantified the pathogen abundance by measuring the copy number of *E. amylovora*-specific gene *amsC* (Fig. 1A). The mono- and co-inoculations of *Pa, Ps* did reduce in average *amsC* gene copy number in comparison to mono-inoculation treatments (Fig. 2A, *Ps:Ea, Pa:Ea, Pa:Ps:Ea* vs *Ea*, respectively); however, these reductions were not significant (Fig. 2A). In contrast, we observed that the application of streptomycin did significantly reduce *Ea* abundance in comparison to the control (Fig. 2A, *Ea* vs *Ea*:strep), but this reduction was not significantly different from mono- and co-inoculation treatments (Fig. 2A, *Ea*:strep. vs *Pa:Ea*, *Ps:Ea,* and *Pa:Ps:Ea*, respectively). These results demonstrate that neither inoculation of *Pa*, *Ps* or both excluded the pathogen *E. amylovora* from colonizing the apple flowers, but these bacteria may impede the optimal growth of *E. amylovora* in the flowers, to a similar level as streptomycin.

Measuring bacterial abundance from the DNA pool, as with quantitative PCR (qPCR) of the *amsC* gene, recovers sequences from dormant and dead cells or even exogenous DNA. Thus, these assays may overestimate the abundance of the target organism. We hypothesized that the co-inoculation may alter the activity of the pathogen. To test this, we performed rRNA gene sequencing (rDNA) and rRNA gene transcript sequencing (rRNA). The rRNA gene represents the abundance of DNA and the rRNA transcripts, as a component of the ribosome, represents transcriptionally active cells. We limited our analysis to the samples treated with water (control), *Ea*, or *Pa and Ps* followed by *Ea* (*Pa:Ps:Ea*). Both *Pa* and *Ps* were detected in the control samples, displaying average relative abundances of 0.75 and 0.22 in the rDNA [Fig. 2B, *Pantoea* amplicon sequence variant (ASV), *Pseudomonas* ASV, control, respectively] and 0.45 and 0.32 in the rRNA (Fig. 2C, *Pantoea* ASV, *Pseudomonas* ASV, control, respectively). This observation reinforces the characterization that these two strains are common members of the apple stigma flora. Additionally, they emerged as significant contributors to the active microbial community, evident from their prevalence in the rRNA data sets (Fig. 2C). In contrast, *Ea* showed minimal presence in the water control samples, accounting for only 0.03 of the rDNA sequences (Fig. 2B, *Erwinia* ASV, control) and only 0.08 of the rRNA sequences (Fig. 2C, *Erwinia* ASV, control). These results suggest that *E. amylovora* naturally occurs with lower abundance within the apple flower microbiota.

The inoculation of *E. amylovora* led to a significant increase in the abundance of the pathogen in both the rDNA and rRNA pools (Fig. 2B and C, *Erwinia* ASV, respectively). Upon inoculation of *Ea,* there was little effect on the relative abundance of *Ps* as there were no significant differences in the relative abundance of the *Pseudomonas* ASV in either rDNA or rRNA pools (Fig. 2B and C, *Pseudomonas* ASV, respectively). Comparatively, inoculation of *Ea* showed a detrimental effect on *Pa*, which decreased significantly in abundance in both rDNA and rRNA pools (Fig. 2B and C, *Pantoea* ASV, *Ea*, respectively), suggesting reduced participation in both the total and the active communities. This trend suggests the possibility of a competitive interaction between the pathogen *E. amylovora* and the flower commensal *Pantoea*.

After the co-inoculation treatment*, Ea* was still readily detected, making up 0.34 of the rDNA and 0.16 of the rRNA pool (Fig. 2B and C, *Erwinia* ASV, *Pa:Ps:Ea*, respectively). Although there was no significant increase in the relative abundance of *Ps* in the rDNA pool upon the co-inoculation treatment, we observed a moderate but still significant increase in the rRNA pool for this bacterium (Fig. 2C, *Pseudomonas* ASV, *Pa:Ps:Ea*). On the contrary, the co-inoculation treatment led to a significant increase in the relative abundance of *Pa* in the rDNA pool, but this increase was less than the water control (Fig. 2B, *Pantoea* ASV, *Pa:Ps:Ea* vs control). In the rRNA pool, we observed a significant increase in *Pa* relative abundance, but this increase was not significantly different from the water control (Fig. 2C, *Pantoea* ASV, *Pa:Ps:Ea* vs control). Our findings suggest that a high inoculation of *E. amylovora* may displace a fraction of the *Pantoea* population, but this effect is overcome, to some extent, by re-inoculating the *Pantoea* strain. Yet, it is important to note that 16S rRNA gene sequencing produces abundances on a relative scale (17). Thus, increasing one member necessitates decreasing another member(s), even though the absolute abundance remains unchanged. This could partly explain why we do not observe significant changes in *E. amylovora amsC* copy numbers in the probiotic inoculation but relatively large reductions in the 16S rRNA/rDNA data sets. These observations reflect a better performance of *Pa* rather than a reduction in *Ea* and that makes up a substantial proportion of both the total and the active populations on the apple stigma. Thus, *Ea* does not appear to be competitively excluded from the stigma after the co-inoculation treatment. However, it is still plausible that the inoculations of the flower commensals decrease the abundance of the pathogen on the flowers.

As a final test to investigate whether the bacterial cells co-occur on the stigma, we visualized the colonization of the stigma of apple flowers by labeled bacteria that actively express fluorescent proteins (FP). Flowers were exposed to *Pseudomonas* CT1181 expressing mCherry FP, *Pantoea* CT-1039 expressing mTurquoise FP, and *E. amylovora* 1189 expressing green FP. Microscopic inspection of these stigmas showed that all three labeled strains actively expressed fluorescent proteins and co-existed in proximal vicinity to each other (Fig. 2D). By visually inspecting the micrographs, we could readily detect that *Pantoea*-mTurquoise, *Pseudomonas*-mCherry, and *E. amylovora*-GFP co-occur at micro-scale on the flower stigma (Fig. 2D). These results further corroborate that the flower commensal bacteria do not competitively exclude the pathogen *E. amylovora*, but they co-occur on the apple flowers, even in close vicinity, and *E. amylovora* remains alive and active as evidenced by expression of the fluorescent marker.

### The pathogen and both flower commensal bacteria co-exist under co-culture growth conditions

**Fig 3:**
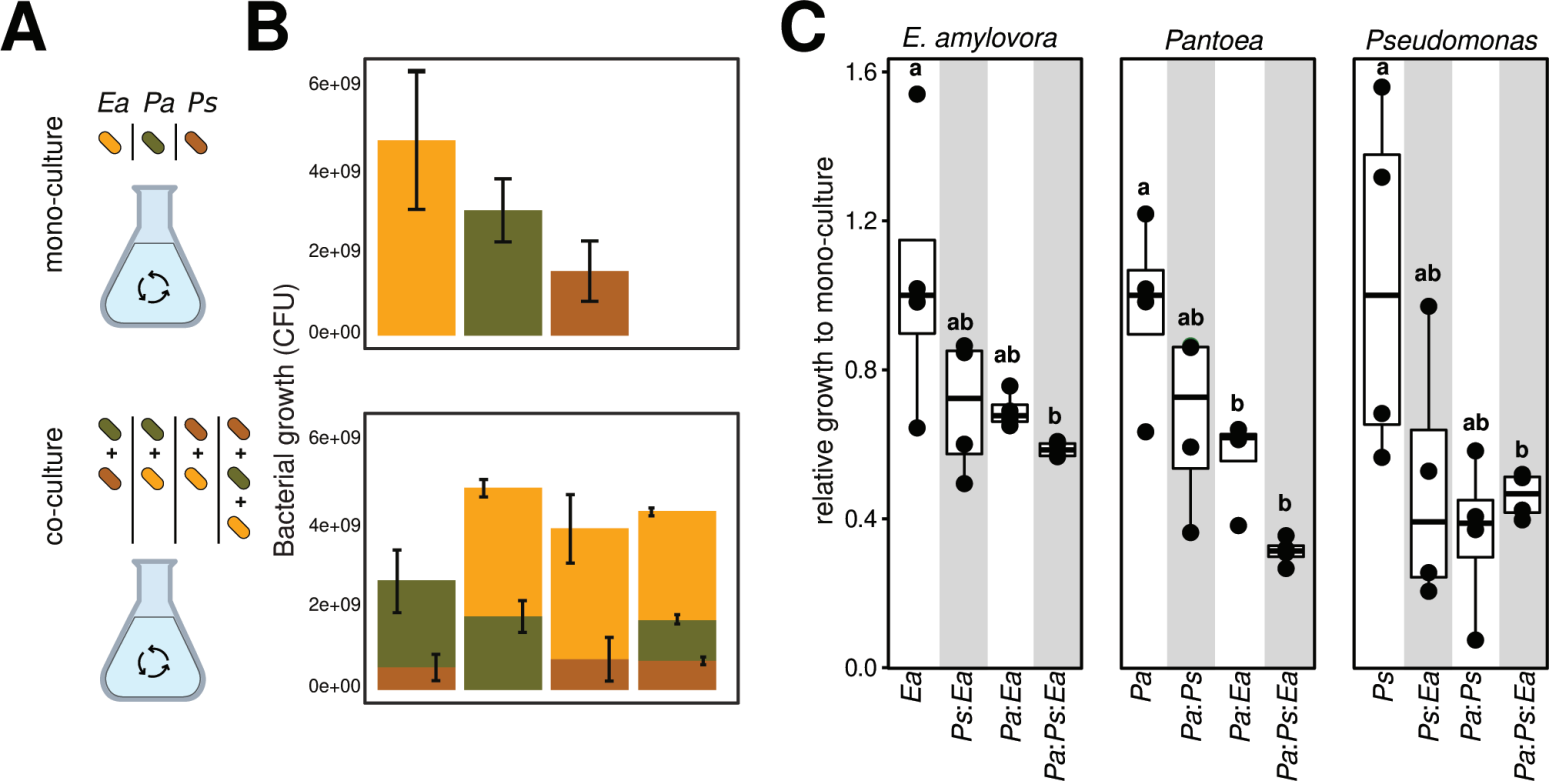
*In vitro* co-culture of the two flower commensal bacteria and the fire blight pathogen. (A) The schematic depicts *in vitro* mono- and co-culture of *E. amylovora* (*Ea*), *Pantoea* (*Pa*), and *Pseudomonas* (*Ps*) using a synthetic stigma exudation medium. (**B)** Bar plots show bacterial CFUs that were determined after 6 h post-inoculation using Lysogeny Broth agar with a selective antibiotic (Materials and Methods). The three bacterial species reached different growth rates in mono-culture, but none of the bacterial strains were out-competed in co-culture. (**C)** The boxplots show the relative growth of each bacterium in co-culture compared to its mono-culture growth condition. A relative growth >1 indicates that the strain grows more abundantly in the co-culture vs mono-culture, whereas a value <1 indicates otherwise. Boxplots labeled *Ea*, *Pa*, and *Ps* show growth variations in the mono-culture of *E. amylovora*, *Pantoea*, and *Pseudomonas*, respectively. Ternary co-culture of the bacteria leads to growth depletion, but none of the strain is out-competed to extinction.

Because both the pathogen (i.e., *E. amylovora*) and the commensal strains (i.e., *Pa* and *Ps*) co-exist on the apple flowers, we wanted to further investigate the interactions between these strains under *in vitro* conditions. Using a synthetic stigma exudate medium (16), we cultured the strains in mono- or co-association (Fig. 3A). After 6 h of growth under shaking, we counted colony-forming units (CFUs) for each strain by using selective antibiotics (Materials and Methods). In mono-cultures, the average cell densities of *Ea* reached 4.8 10^9^ CFUs/mL, whereas *Pa* and *Ps* reached an average of 3.1 10^9^ and 1.6 10^9^ CFUs/mL, respectively (Fig. 3B, top panel). These results suggest that each strain reached similar population densities in the synthetic media, although *Ea* had the largest population size, followed by *Pa* and *Ps*. In pairwise and ternary co-cultures, the total bacterial density reached on average 2.68 10^9^, 3.95 10^9^, 4.37 10^9^, and 4.94 10^9^ CFUs/mL in *Pa:Ps*, *Ps:Ea*, *Pa:Ps:Ea*, and *Pa:Ea* co-cultures, respectively (Fig. 3B, bottom panel), suggesting similar cell densities were reached across the co-cultures. Importantly, none of the tested strains were competitively excluded in pairwise or ternary co-culture conditions. However, we noted a decrease in the relative growth of *E. amylovora*, *Pantoea,* and *Pseudomonas* upon ternary co-culture compared to the mono-culture (Fig. 3C, *Pa:Ps:Ea*). Together with the observations made in Fig. 2, these results demonstrate that these bacteria co-exist under *in vivo* and *in vitro* growth conditions. In fact, *Ea* consistently reached higher cell densities than the other strains in these conditions, providing further evidence that *E. amylovora* is not competitively excluded and co-exists with the two flower commensal bacteria.

### Ternary inter-species interactions lead to the emergence of new expression patterns

**Fig 4:**
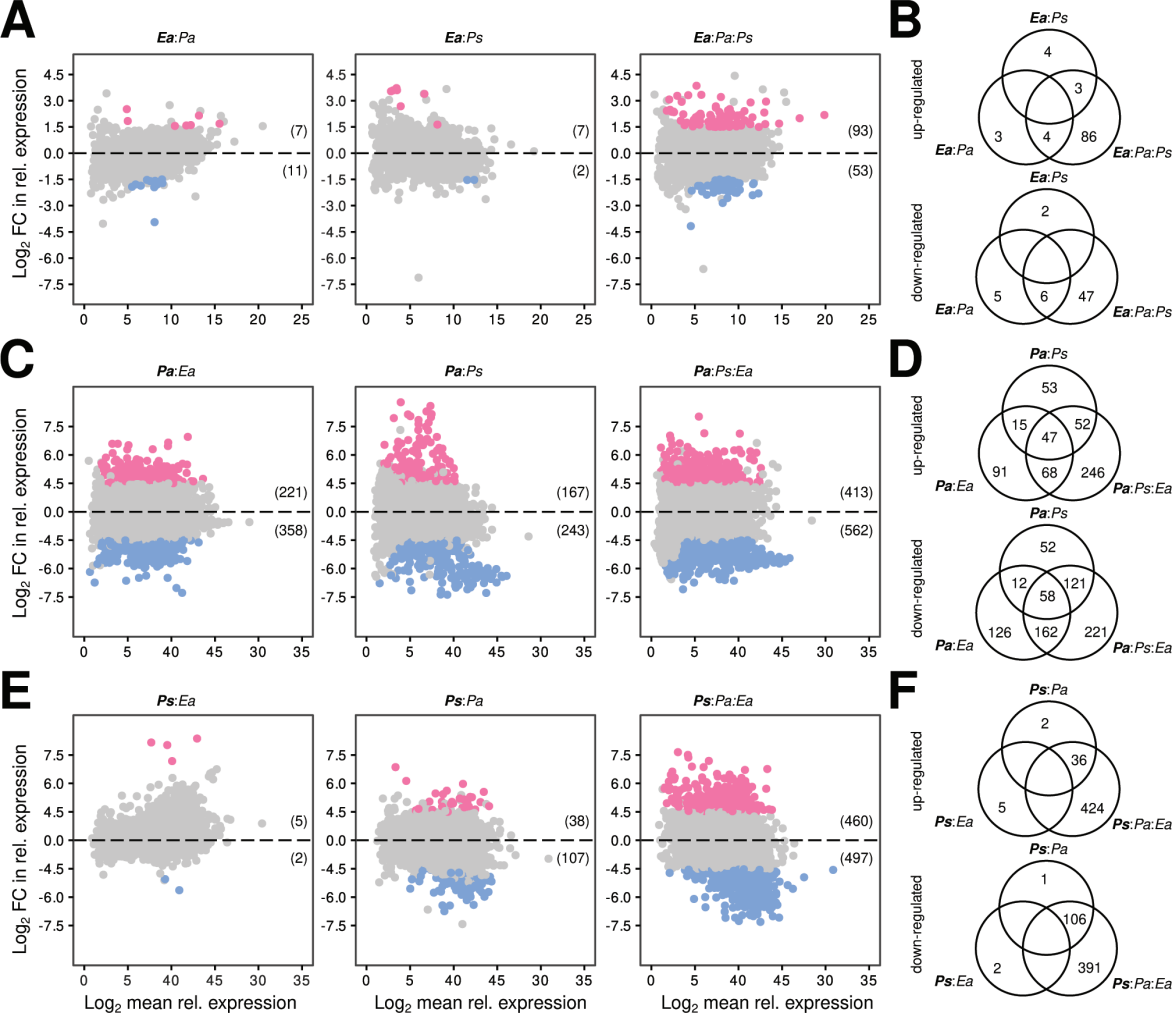
Co-culture of the three bacterial strains leads to new expression patterns. (A, **C,** and** E)** depict MA plots (log fold-change versus mean expression) showing DEGs in *Ea*, *Pa*, and *Ps* upon binary and ternary co-culture, respectively. Count data were fitted to negative binomial distribution (DESeq2) and DEGs were determined based on the cutoffs of 1.5 and 0.05 in log_2_ fold change expression and Benjamini-Hochberg adjusted *P*-value, respectively. Red and blue indicate significantly up- and down-regulated genes, and gray indicates no significant change in the expression. Numeric values indicate a total number of genes up- or down-regulated. Ternary co-culture of *Ea*, *Pa,* and *Ps* led to more DEGs in the three strains compared to their binary co-culture. (**B**,** D,** and** F)** show Venn charts indicating the number of DEGs shared between binary and ternary co-cultures in *Ea*, *Pa*, and *Ps*, respectively. Top Venn diagrams in (**B**, **D**, and **F) **correspond to significantly up-regulated genes *Ea*, *Pa,* and *Ps* respectively. Bottom Venn diagrams correspond to significantly down-regulated genes in *Ea*, *Pa,* and *Ps,* respectively. In *Ea* and *Ps*, we noted no core genes significantly up- or down-regulated shared between all co-culture growth conditions. *Pa* showed a small fraction of core genes significantly up- or down-regulated shared between all co-culture conditions.

Next, we sought to understand how the co-culture of *Pa*, *Ps,* and *Ea* modulate gene expression in these three strains. We performed transcriptome analysis for the three strains in different strain assemblages including mono-cultures and binary and ternary cultures of *Pa*, *Ps,* and *Ea* (Fig. 3A). To quantify the changes in gene expression, we computed differentially expressed genes (DEGs) by contrasting relative gene expression in co-cultures versus mono-culture growth conditions (Materials and Methods). The ternary co-culture combination (Fig. 4A, C, and E, 3rd panel) led to more DEGs than any binary co-culture growth conditions (Fig. 4A, C, and E, first and second panels) in all three strains tested. For example, we observed 146 DEGs (93 genes with increased expression and 53 genes with decreased expression) in *E. amylovora* grown in a ternary mixture compared to when *Ea* was grown in mono-culture (Fig. 4A, third panel). In contrast, binary co-culture of *Ea* with *Pa* or *Ps* led to only 18 and 9 DEGs of combined up- and down-regulated genes, respectively (Fig. 4A, first and second panels). Similar patterns were observed for *Ps and Pa*, with larger DEGs in ternary cultures than binary co-cultures (Fig. 4C and E, respectively). None of the DEGs in *Ps* and *Ea* were shared across all co-culture growth conditions (Fig. 4B and F, respectively). However, we noted 47 up-regulated and 58 down-regulated genes in *Pa* that were differentially regulated across all co-culture growth conditions (Fig. 4F). These findings highlight that the differentially expressed genes were not additive or predictable from binary interactions. Instead, the ternary inter-species interactions led to the emergence of new expression patterns for each member of this community.

**Fig 5:**
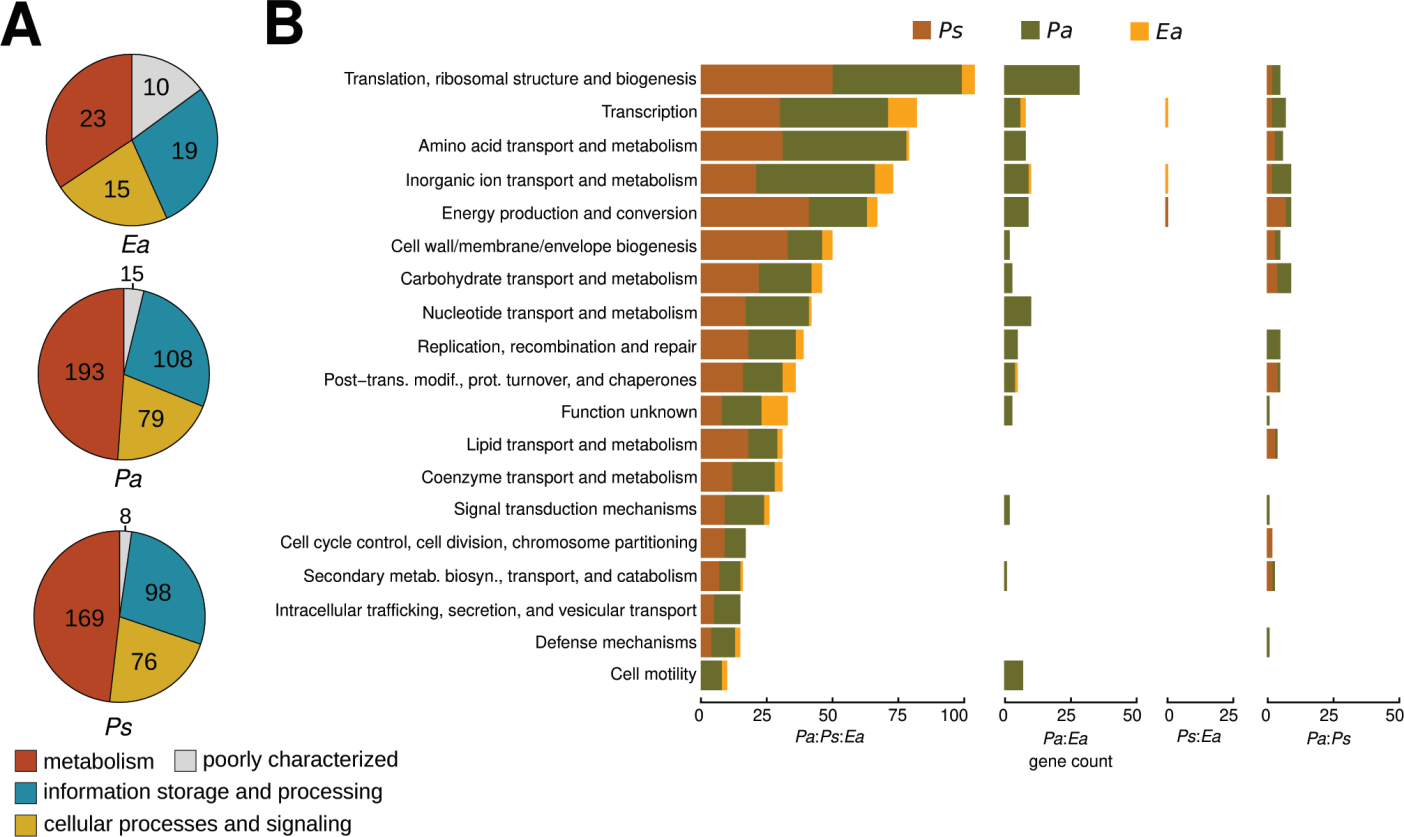
Functional annotation of differentially regulated genes. (A) shows circle chart that depicts the proportion of the functional annotation of DEGs in *Ea* (top), *Pa* (middle), and *Ps* (bottom). Color code indicates category, and values inside the chart indicate gene counts. More DEGs are related to metabolism, than to information storage and processing, or cellular processes and signaling. (**B**) Barplots indicate the classes of COGs of the annotated DEGs in the three strains. Color denotes bacterial strain. Ternary co-culture results in the increase of DEGs and annotated COGs classes.

To characterize the functions associated with multi-strain interactions, we functionally annotated and classified DEGs to Clusters of Orthologous Genes (COGs) (18, 19). The majority of DEGs in *Ea* (79 genes)*, Pa* (395 genes), and *Ps* (351 genes) were not assigned to a molecular function or a biological process (Table S1). Among DEGs that can be assigned with known functions, most of these were metabolism-related genes with 34.3% or 23 DEGs in *Ea*, 48.8% or 193 DEGs in *Pa,* and 48.1% or 169 DEGs in *Ps* (Fig. 5A). Other DEGs identified belong to the categories of information storage and processing and cellular processes and signaling. Further characterization of the DEGs revealed that these genes cover a wide range of specific biological functions and processes (Fig. 5B). Importantly, several of the DEGs functions, such as intracellular trafficking and secretion and coenzyme transport and metabolism, were exclusively identified during *Pa:Ps:Ea* ternary interactions but not in any binary interactions. Other functions such as transcription or energy production and conversion were more represented in ternary interactions as compared to binary interactions (Fig. 5B). Notably, several DEGs were involved in the transport and metabolism of amino acids, inorganic ion, carbohydrate, nucleotide, and lipid in both commensal bacteria and the pathogen (Fig. 5B). We also identified DEGs belonging to ABC transporters (Fig. S1A), secretion systems (Fig. S1B), and prokaryotic defense systems (Fig. S1C). The gene set enrichment analysis of DEGs showed the over-represention of 19, 160, and 150 Gene Ontology (GO) terms in *Ea*, *Pa* and *Ps*, respectively (Table S2), whereas only 11 Kyoto Encyclopedia of Genes and Genomes (KEGG) metabolic pathways were over-represented in the three bacterial strains (Table S3). To determine potential behavior changes of *Ea* during ternary interactions as compared to binary and mono-culture, we further examined functions of individual DEGs uniquely identified in *Ea* during ternary interactions (Table S1). Notably, two DEGs with functions related to virulence regulation were identified: *slyA* (Ea_01648) (20) and *rcsB* (Ea_02226) (21). Four DEGs related to motility were identified: sigma factor *fliA* (Ea_02027), flagellar regulator *flk* (Ea_02294), *fliS* (Ea_02031), and flagellar hook-associated protein 1 (Ea_01424). Six DEGs related to antimicrobial resistance/tolerance were identified: tripartite efflux system *emrA* (Ea_01261) and *emrB* (Ea_01390), fosmidomycin resistance protein (Ea_01027), blue copper oxidase CueO (Ea_00767), multidrug efflux pump accessory protein *acrZ* (Ea_01184), and outer membrane protease *ompP* (Ea_00731). Finally, we also identified nine DEGs related to stress response: *clpB* (Ea_02585), *apaG* (Ea_00661), peptide methionine sulfoxide reductase *msrB* (Ea_01894), phage-shock-protein (*psp*) operon transcriptional activator (Ea_01805), organic hydroperoxide resistance transcriptional regulator (Ea_03382), DNA damage-inducible protein I (Ea_01405), heat shock protein *hspQ* (Ea_01362) and *ibpA* (Ea_03427), and cold shock-like protein *cspE* (Ea_01091). Interestingly, several DEGs identified in *Ea* during ternary interactions are related to tolerance to acidic conditions, such as spermidine export protein *mdtJ* (Ea_02894) (22) and regulator of acid tolerance *rcsB* (Ea_02226) (21), which suggests a possible adaptation of *E. amylovora* to reduced pH in the growth milieu.

Several DEGs identified in *Pa* and *Ps* upon ternary co-culture are involved in microbe-microbe interactions (Table S1). These include toxin/antitoxin system genes (*hicB* Pa_01959, *relB* Pa_04496, *relE* Pa_04497, *higB-1* Pa_02515, *higB-2* Ps_01321, *hipA* Ps_03571, and *fitB* Ps_01790), quorum sensing (Ps_05074), iron utilization (Ps_03966), and biofilm formation (*bdlA* Pa_03522 and Ps_02062), suggesting modulation of genes involved in inter-species interactions.

Taken together, our meta-transcriptomic analysis shows that the three bacterial strains undergo substantial transcriptional re-programming in response to ternary inter-species interactions and highlight the emergence of microbial functions that were not identified in mono- and binary co-cultures.

**Fig 6:**
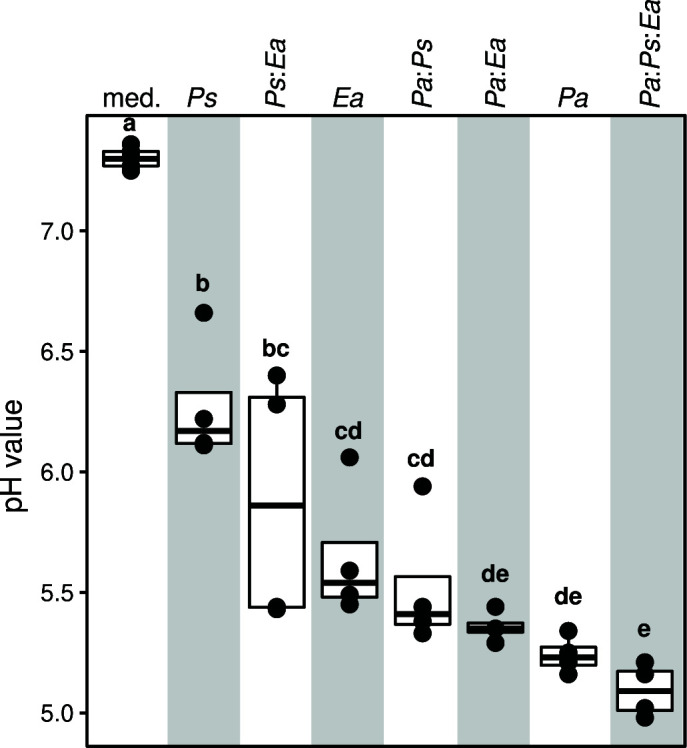
Ternary co-culture leads to strong acidification of the pH. The boxplots show pH values in the control (no bacterial inoculation), the mono- and the co-cultures. Inoculation of any of *Ea*, *Pa*, *Ps* or their combinations significantly alters the pH of the growth milieu. Ternary co-culture of the bacteria shifts the pH to more acidic than any binary co-culture or mono-culture. Color denotes growth condition, and letters show significance in the *P*-value (*P* < 0.05, Kruskal-Wallis test followed by Conover’s *post hoc* test).

Finally, to test if co-culturing influenced the external environment, we measured the pH at the end of bacterial growth. We detected pH reductions in all growth conditions (Fig. 6). Mono-cultures of *Ps*, *Ea,* and *Pa* led to a reducing pH on average from 7.30 (media control) to 6.27, 5.64, and 5.24, respectively (Fig. 6). We noted that any binary co-culture with *Pa* (i.e., *Pa:Ea* and *Pa:Ps*) led to more acidic medium than the binary co-culture of *Ps* and *Ea* (Fig. 6). Notably, the pH of the ternary co-culture was even more acidic than any of the binary or mono-culture conditions (Fig. 6). Thus, while the growth of all three strains alters the pH of the milieu, the ternary co-culture of *Ea*, *Pa,* and *Ps* strongly reduced the pH, pointing to potential interactive effects of these strains in co-culture, and another emergent property of the ternary growth conditions.

## DISCUSSION

In this study, we showed that the co-inoculation of two members of the flower microbiota, *Pseudomonas* CT-1059 and *Pantoea* CT-1039, reduced fire blight infection caused by *Ea* in experimental orchards (Fig. 1B). These results corroborate previous finding (15) and further point to the potential use of these strains as probiotic inoculants to control fire blight. Several bacterial strains that control fire blight have been previously identified and used, such as *Pseudomonas fluorescens* A506 (23)*, Bacillus subtilis* QST713 (24), *Pantoea agglomerans* E325 (25), and *Pantoea vagans* (26), to cite only a few. The mechanism by which the above-mentioned biocontrol bacteria protect the plant from the pathogen is attributed to their ability to reduce or suppress the growth of *E. amylovora* on the stigma through antibiosis or competing for nutrients and space (11, 27, 28). In contrast, our study did not provide evidence of competitive niche exclusion of *E. amylovora* by the commensal strains. The abundance of the pathogen remained relatively high on the flower stigma, fluorescence micrographs showed that *E. amylovora* co-localize at the micro-scale with both flower commensals, and all three strains co-existed in the same culture media (Fig. 3B). Therefore, these bacterial strains do not seem to inhibit the establishment of *E. amylovora* populations; instead, these results indicate the ecological co-existence among the three bacteria on flowers.

Nevertheless, we did observe smaller *Ea* populations both through *amsC* copy numbers (Fig. 2A) and 16S rRNA gene/transcript sequencing (Fig. 2B and C) in the presence of the bacterial strains, suggesting potentially reduced population sizes of *E. amylovora*. Our findings cannot rule out the possibility that these flower commensal bacteria restrict the population size of the pathogen below a threshold that impairs its pathogenicity or infectivity. Future work will help us test this hypothesis and reveal how *Pseudomonas* and *Pantoea* alter *E. amylovora* population on the stigma of apple flowers.

As the commensal bacteria did not appear to significantly hinder the growth of *E. amylovora*, either *in vivo* on the stigma of apple flowers (Fig. 2A) or *in vitro* in synthetic media (Fig. 3B), our next objective was to investigate whether these strains influence the activity of the pathogen. We postulated that the presence of the commensal flower bacteria affects the activity of *E. amylovora* and their interactions lead to emergent properties translated by gene expression. An emergent property is defined here by the emergence of functions or phenotypes not predictable from the constituent part of the community when observed in isolation (6, 29). The meta-transcriptome study revealed changes in the transcription profiles of all three bacteria when in ternary co-culture. These expression patterns were unique compared to when they were grown alone, or even in binary co-cultures (Fig. 4A, C, and E). Characterization of DEGs identified in ternary interactions revealed that these genes covered various microbial functions and processes (Fig. 5B; Table S2). Importantly, several of the DEGs encoded for molecular functions that mediate bacterial inter-species interactions, ABC transporters, defense mechanisms, or stress response (Fig. S1; Table S1). These findings indicate that the two flower-associated bacteria and the floral pathogen undergo substantial re-programming in gene expression under ternary interactions, and the emergence of microbial functions is non-additive to the binary interactions.

It is reasonable to hypothesize that the transcriptional re-programming of *E. amylovora* during ternary co-culture (146 DEG/3,674 total genes, or 4% total genes) leads to phenotypical changes in the pathogen. From our analysis, we identified two genes that may play an important role in the virulence of *E. amylovora* or related bacteria. First, RcsB (Table S1, Ea_02226, log_2_FC = 1.53, *P*-value = 3.71E-05) is a response regulator of the RcsBCD phosphorelay system that is a major regulator of the biosynthesis of exopolysaccharide and amylovoran in *E. amylovora* (30). Amylovoran is known to be critical for biofilm formation in *E. amylovora* (31) and has been suggested to mediate a protective role against plant defense during infection (7). Second, SlyA (Ea_01648, log_2_FC = 2.45, *P*-value = 1.54E-11) is a virulence regulator of the type III secretion system in closely related bacterial plant pathogen *Dickeya dadantii* (20). The altered expression of this regulator gene may impact the ability of *E. amylovora* to infect its host. Furthermore, to cause an effective infection, *E. amylovora* is required to migrate to the base of the flower that harbors natural openings. For this, motility is required. In ternary co-culture, the expression of several motility-related genes is suppressed in *E. amylovora* (e.g., *fliA* Ea_01648, log_2_FC = −1.75, *P*-value = 0.03). Furthermore, up-regulation of several genes involved in stress responses and antimicrobial tolerance (e.g. multidrug efflux pump gene *acrZ*, Ea_01184, log_2_FC = 2.23, *P*-value = 2.15E-06) was observed during ternary co-culture. These results suggest that the fire blight pathogen may be under stress when interacting with the commensal flower bacteria. Although we could not attribute the suppressed disease infection phenotype to DEGs, the transcriptomic re-programming of *Ea* of multiple DGEs during the ternary interaction could be one explanation for the reduced infection upon interactions with *Ps* and *Pa*. Regarding the commensal bacteria *Ps* and *Pa*, DEGs with various functions related to microbe-microbe interactions, such as quorum sensing, biofilm formation, toxin production, and nutrient acquisition, were identified in ternary interaction but not in mono- or binary interactions (Table S1), suggesting enhanced microbe-microbe interaction activities during ternary interaction, which suggests these bacteria also modulate their behavior in the presence of *Ea*.

Our analysis of the gene expression data pointed to metabolic or physiological changes in *E. amylovora* indicative of adaptations to an acidified environment and general stress response in both commensal bacterial strains (Table S1). *Pantoea* was the strongest driver of pH reduction, and ternary co-culture led to the strongest pH acidification of the growth milieu (Fig. 6). It is noteworthy that acidification has previously been reported as a mechanism by which biological control strains may reduce *Erwinia amylovora* pathogenicity. Pusey and colleagues have shown that *Pantoea agglomerans* strain E325 acidifies the pH of the flowers to a point that could deplete the growth of the fire blight pathogen (25). Furthermore, Pester and colleagues have shown that reducing the pH of apple flowers leads to the down expression of virulence genes in *E. amylovora* (32). Similar dynamics have been described for other pathogen-host systems. For instance, the bumble bee symbiont *Lactobacillus bombicola* was shown to inhibit the pathogen *Crithidia bombi* by reducing the pH in the gut (14). These studies highlight the role of environmental pH as a potential property that modulates microbial interactions and potentially pathogen behavior (33, 34). It remains to be tested whether co-inoculation of *Pseudomonas* and *Pantoea* could lead to down-regulation of pathogenicity genes in *E. amylovora* and whether this is a direct consequence of acidified habitat on the apple flower.

In conclusion, this study showed that co-inoculating two flower-associated bacteria protects the host plant from the fire blight disease. These two bacterial strains did not competitively exclude *E. amylovora* from the stigma habitat, but bacterial inter-species interactions led to alterations in the activities of all three members. Ternary interactions between the bacteria led to a strong alteration of the pH beyond what was expected from any strain alone and to the emergence of new gene expression patterns. Our study emphasizes the role of emergent microbial properties that are not predictable from binary interactions. We propose further exploring inter-species interactions between microbial consortia and plant pathogens to mediate disease resistance. As the outcome of inter-species interactions is inherently complex, microbial virulence may be an emergent microbial propriety itself (35). In this respect, deciphering these interactions requires empirical testing and needs to be understood in their ecological context. Exploring the properties of microbiota commensals offers an alternative and sustainable practice to manage plant diseases and promote host health.

## MATERIALS AND METHODS

### Bacterial strains and growth conditions

Bacterial strains used in this study were *Erwinia amylovora* strain 110, *Pseudomonas* CT-1059 (field co-inoculations and *in vitro* co-culture assay), *Pantoea* CT-1039 (field co-inoculations, *in vitro* co-culture assay and microscopy imaging), and *Pseudomonas* CT1181. All strains were cultured in Lysogeny Broth (LB) medium at 28°C overnight with shaking (250 rpm). Antibiotics were added at the following concentrations when required: chloramphenicol, 30 µg/mL; kanamycin, 50 µg/mL; ampicillin, 100 µg/mL; spectinomycin, 50 µg/mL.

### Bacterial strain co-inoculations in the field and fire blight scoring

Field co-inoculations were performed on 30-year-old apple tree cultivar “Red Delicious” in May 2021 at the Lockwood Farm, Hamden, CT (41.406 N, 72.906 W). Thirty-six apple trees were randomly grouped into nine treatment groups with four tree replicates per treatment and organized in a randomized complete block design. *Pantoea* CT-1039 and *Pseudomonas* CT-1059 were cultured in LB overnight. Cells were pelleted by centrifugation at 5,000 rpm for 15 min, adjusted to ca. 5 × 10^7^ CFU/mL in water, and then sprayed to apple flowers approximately 2 L per tree. Treatments were applied twice, at 60% bloom (29 April 2021) and at 80% bloom (30 April 2021), using a motorized Solo sprayer (3.8 L per tree). *E. amylovora* was inoculated at 100% bloom (1 May 2021) with the concentration of 5 × 10^6^ CFU/mL. Streptomycin (Firewall 50, at 100 ppm) was applied 2 h post *Ea* inoculation (1 May 2021). For *Pa:Ps* co-inoculation, the bacteria were mixed at equal volume and applied to the same dosage as single strain treatments. Fire blight disease infection was rated 3 weeks after the inoculation of *E. amylovora*. Disease incidence was calculated as percentage of symptomatic flower clusters from the pool of treated flower clusters of each tree per treatment.

### Flower sampling for *E. am*ylovora quantification

From the field co-inoculation experiments conducted May 2021, we collected flower samples for DNA and RNA extractions. DNA from the single flower was used to quantify *E. amylovora*, and to profile microbial communities using 16S rRNA gene, whereas RNA from 20 flowers were used to profile the flower microbiota using 16S rRNA gene transcripts. Flowers were harvested after 2 days post-inoculation of *E. amylovora* (3 May). For DNA isolation, 10 individual flowers for each treatment were harvested. Four to five stigmas from each flower were dissected, placed into micro-centrifuge tube using sterile scissors, snap frozen in liquid nitrogen, and were treated as a single biological replicate. For RNA extraction, stigmas were collected from 20 flowers, placed into micro-centrifuge tube, snap frozen, and treated as one biological replicate. Samples were stored at −80°C until further processing.

### DNA extraction from apple stigma

Deep-frozen stigma samples were retrieved from −80°C and 200 µL of 1× phosphate-buffered saline (PBS) (pH 7.4, Invitrogen, Carlsbad, CA) + 0.001% Silvet L77 (PlantMedia, Dublin, OH) were added to the tube. To retrieve epiphytic bacteria from the stigma surface, samples were sonicated for 5 min in a water batch at room temperature followed by full-speed vortex for 30 s. Stigma tissues were removed from the tube and DNA was extracted from the solution using DNeasy PowerSoil Pro Kit (Qiagen, Hilden, Germany) following manufacturer’s instructions, except the following modifications. Cell suspensions were treated with 36 µL of lysozyme (10 mg/mL in 1× PBS, AmericanBio, Natick, MA) for 30 min at 37°C followed by 20 µL of proteinase K (>600 U/mL, ThermoFisher Scientific, Waltham, MA) and 5 µL of RNase A/T1 (2 mg/mL / 5,000 U/mL, Fisher Scientific, Waltham, MA) for 5 min at room temperature. DNA was eluted in 35 µL of nuclease-free water (Qiagen, Hilden, Germany).

### RNA extraction from apple stigma

Deep-frozen stigma samples were retrieved from −80°C and 200 µL of 1× PBS (pH 7.4, Invitrogen, Carlsbad, CA) + 0.001% Silvet L77 (PlantMedia, Dublin, OH) + beta-mercaptoethanol (% vol/vol) were added to the tube. Epiphytic bacteria were detached by combining water bath sonication for 5 min and followed by full-speed vortex for 30 s as described in the DNA extraction protocol. Stigma was removed from the tube, and RNA was extracted using the RNeasy PowerLyzer Tissue & Cells Kit (Qiagen, Hilden, Germany) according to the manufacturer’s instructions. Yield and quality were determined using QuBit and Agilent Bioanalyzer, respectively. Eluted RNA was depleted from ribosomal RNA using NEBNext rRNA Depletion Kit (Bacteria, New England Biolabs, MA), then used as a template to generate cDNA using NEBNext Ultra RNA Library Prep Kit for Illumina (England Biolabs, Ipwich, MA). The resulting cDNA was assessed with a high-sensitivity Agilent 2100 bioanalyzer. Size profiles of cDNA fragments were consistent across samples.

### Quantification of *E. amylovora* by qPCR

The absolute abundance of *E. amylovora* in each stigma sample was quantified using a previously described method (36). In brief, the DNA copy number of *E. amylovora* was quantified by determining the cycle threshold (CT) value of the *E. amylovora*-specific gene *amsC*. qPCR was performed using SsoAdvanced universal SYBR Green supermix (Bio-Rad, CA, USA) on a Bio-Rad CFX96 real-time PCR, as described previously (37). The CT values for a 1/10 dilution series of known *amsC* gene copies of *E. amylovora* chromosomal DNA were determined to make a standard curve for calculating copy numbers in stigma samples.

### Confocal microscopy observation of microbial colonization on stigma

*Pseudomonas* CT-1181, *Pantoea* CT-1039 constitutively expressing mCherry (red), and mTurquoise (turquoise) FP were generated using miniTn7-pURR25DK-3xmCherry and miniTn7-pURR25DK-mTurquoise, respectively (38). Using tri-parental mating including helper strain *Escherichia coli* RHO3 carrying pTNS, mCherry, and mTurquoise were inserted at unique site *att*Tn7 in the genome of recipient *Pseudomonas* CT-1181 and *Pantoea* CT-1093, respectively (39–41). Derivative *E. amylovora* 1189 expressing green fluorescent protein carried a *gfp* (green) under the control of *nptII* promoter integrated into the chromosome. Bacterial cells were cultured in LB overnight at 28°C shaking (250 rpm). Cells were pelleted by centrifugation and adjusted to 10^7^ CFUs/mL before spray-inoculating onto the stigma of 3-year-old trees of the cultivar “Gala.” Trees were potted in separate containers. Inoculated trees were kept in a plant growth chamber (25°C, 80% relative humidity, and 16-h cycle of 350 µmol light intensity). *Pseudomonas* CT-1181 expressing mCherry FP and *Pantoea* CT-1039 expressing mTurquoise FP were inoculated on stigma when petals first open, and *E. amylovora* 1189 expressing green FP was inoculated 1 day thereafter. Stigmas were collected 2 days post the inoculation of *E. amylovora*. Fluorescent green (484 nm/507 nm), mCherry (587 nm/610 nm), and mTurquoise (434 nm/474 nm) signals were observed with a Leica TCS SP5 confocal microscope (Leica Microsystems, Wetzlar, Germany) equipped with four laser channels (405 nm, multiline Argon, 561 nm and 633 nm) and two HyD detectors. Images were captured with Leica LAS AF software and overlayed using Leica LAS-X software.

### Profiling the microbiota of apple flower stigma

To study the relative abundance of *E. amylovora*, *Pseudomonas,* and *Pantoea* on the apple flowers, we profiled the flower microbiota using a previously described protocol (3, 35). Briefly, the V4 region of the 16S rRNA gene was amplified using primer set, 515F and 806R in triplicate from either isolated DNA or generated cDNA. PNA clams were added to PCR mixture to reduce the amplification of apple plastids and mitochondrial sequences (3). PCR products from the triplicates were pooled, purified, and normalized using SequalPrep kit (Invitrogen, Carlsbad, CA). Normalized amplicons were pooled together and submitted for sequencing using MiSeq v2.2.0 platform (Illumina Inc, San Diego, CA) at Yale Center for genome analysis.

### Sequences processing and community analysis

MiSeq forward and reverse reads were joined and demultiplexed using qiime2 pipeline (q2cli v2021.4.0) (42). PhiX and chimeric sequences were filtered out using qiime2-DADA2 (43). Scripts used for sequence processing are available under https://github.com/hmamine/PPE/tree/main/reads_processing and raw sequencing reads are accessible at sequence read archive accession number PRJNA966219. Taxonomic classification of reads was performed using qiime2 feature classifier (q2cli v2021.4.0) (42) and on scikit-learn naive Bayes trained classifier (44). The classifier was trained on 16S rRNA sequences (Green Genes 13.8.99) using the primer set 515F/806R. Reads were assigned to ASVs with 100% sequence identity. All R scripts used in this study are indicated at https://github.com/hmamine/PPE.

### *In vitro* mono- and co-culture growth assays

For mono-culture assay, cells from 5 mL of an overnight culture of *E. amylovora* strain 110, *Pseudomonas* CT-1059, or *Pantoea* CT-1039 were pelleted, washed, and re-suspended in equal volume of 0.5× PBS. Cell concentration was adjusted to an initial Optical Density 600nm (OD_600_) of 0.08 in partial stigma-mimicking media. Cells were incubated at 28°C for 6 h with shaking. For co-culture assays, bacterial cell suspensions were prepared similarly to mono-culture growth assay. Mono-cultures of each strain were adjusted to the same density and mixed in equal volume to produce an initial OD_600_ of 0.08. The population of each strain and the pH of the culture were determined after 7 h of growth. The colony-forming units of each isolate were determined by plating on an LB agar medium with selective antibiotics. *E. amylovora* and *Pseudomonas* CT-1059 were resistant to rifampicin and streptomycin, respectively. *Pantoea* CT-1039 was sensitive to both aforementioned antibiotics. Thus, *Pa* counts were determined by counting cells resistant to both antibiotics and subtracting those counts from cells resistant to either antibiotic independently.

### RNA extraction *in vitro* co-culture assays

Bacterial cells were collected from mono- and co-cultures after 6 h of growth at 28°C in a partial stigma-mimicking medium. RNA was extracted using the RNeasy PowerLyzer Tissue & Cells Kit (Qiagen) according to the manufacturer’s instructions. The RNA was eluted in 50 µL nuclease-free water. Quantity and quality control of the RNA was assessed by using Qubit RNA Broad-Range Assay (Invitrogen) and with RNA ScreenTape analysis on Agilent TapeStation Bioanalyzer. Before Illumina library preparation, rRNA depletion was conducted using the NEBNext rRNA Depletion Kit (Bacteria, New England Biolabs) according to the manufacturer’s recommended protocol. Then, DNA removal coupled with cDNA synthesis was conducted using NEBNext Ultra II RNA Library Prep Kit for Illumina (New England Biolabs) according to the manufacturer’s recommended protocol. DNA quantity was checked using the Qubit dsDNA HS Assay Kit (Invitrogen). Library sequencing was conducted on an Illumina NovaSeq platform through services provided by the Yale Center for Genome Analysis.

### Transcriptome analysis

Adapter removal and sequence trimming were conducted using Trimmomatic (45). The full genome of each of *E. amylovora* 110, *Pseudomonas* sp. strain CT-1059, and *Pantoea* sp. strain CT-1039, available in the GenBank Sequence Read Archive (SRA) under the BioProject accession number PRJNA693803, was concatenated to build a unique index for mapping the reads. The index was generated using Salmon (v1.10) (46). Reads were quantified against the generated index using the alignment tool Salmon (v1.10). Differential expressed genes were identified using DESeq2 R package (47) using normalized reads indicated by transcripts per million. Genomes were annotated using Prokka annotation tool (v.1.14) (48) and the Functional Annotation and Classification of Proteins of Prokaryotes tool (19). The enrichment analysis was computed using the Functional Analysis and Gene Set Enrichment for Prokaryotes tool (19). All scripts are available at https://github.com/hmamine/PPE/tree/main/metatrans.

## Data Availability

All sequencing reads used in this study are available at the NCBI Sequence Read Archive BioProject number PRJNA966219. All R scripts used to generate the figures could be accessed at https://github.com/hmamine/PPE.

## SUPPLEMENTAL MATERIAL

Figure S1 mbio.00213-24-s0001.eps Differentially expressed genes.

Table S1 mbio.00213-24-s0002.xlsx Genes that were differentially expressed in *Erwinia amylovora*, *Pantoea* CT-1039, and *Pseudomonas* CT-1059 in ternary co-culture.

Table S2 mbio.00213-24-s0003.xlsx GO term enrichment analysis.

Table S3 mbio.00213-24-s0004.xlsx KEGG pathway enrichment analysis.

ASM does not own the copyrights to Supplemental Material that may be linked to, or accessed through, an article. The authors have granted ASM a non-exclusive, world-wide license to publish the Supplemental Material files. Please contact the corresponding author directly for reuse.
